# Postbiotics in Functional Foods: Production, Delivery, Preservation, and Regulation

**DOI:** 10.3390/foods15142434

**Published:** 2026-07-09

**Authors:** Niyaz Ali-Haneef, Amar R. Mohite, Punitha Muruganantham, Adhil Anver Salim, Khanita Suman Chinannai, Anish John, Inamul Hasan Madar

**Affiliations:** 1 Centre for Integrative Omics Data Science (CIODS), Yenepoya (Deemed To Be University), Mangalore 575018, Karnataka, India; niyazalinettana@gmail.com (N.A.-H.); punitham.biosci621@gmail.com (P.M.); adhilanver@gmail.com (A.A.S.); sumankhanita6@gmail.com (K.S.C.); anishjohnjj@gmail.com (A.J.); 2 Krishna Institute of Science and Technology, Krishna Vishwa Vidyapeeth “Deemed To Be University”, Karad 415539, Maharashtra, India; dkbiosci@gmail.com

**Keywords:** postbiotics, thermostability, microencapsulation, standardization gaps, regulatory frameworks, biopreservation

## Abstract

Postbiotics—defined by the International Scientific Association of Probiotics and Prebiotics (ISAPP) as preparations of inanimate microorganisms and/or their components that confer a health benefit to the host—are attractive from a stability perspective compared to live probiotics. They withstand thermal processing and room-temperature transport and storage and are compatible with low-pH or low-water-activity food products. Despite the promise, the literature is scattered, with no review that integrates evidence from the development pipeline. This review fills that gap. The ISAPP 2021 definition is reviewed, including the practical difficulty resulting from the exclusion of cell-free supernatant (CFS)-based products that represent most of the experimental evidence. Fermentation-based production systems and non-thermal inactivation technologies—high-pressure processing (HPP), pulsed electric fields (PEF), ultrasound, cold plasma, and supercritical CO_2_—are compared; non-thermal inactivation preserves the activity of thermolabile bacteriocins and phenolic fractions. Delivery systems such as spray-drying, alginate hydrogel microencapsulation, liposomal nanoencapsulation, and carboxymethyl cellulose (CMC) active packaging are assessed for gastrointestinal survival and food system compatibility. Biopreservation potential is reviewed in meat, seafood, dairy, fresh produce, and fermented foods. The regulatory framework for the United States, European Union, Japan, and India is critically reviewed; “postbiotic” is not an explicitly defined term in 2025. Three priority translational bottlenecks are identified: the absence of standardized potency assays, the lack of cross-class quality benchmarks, and the unresolved conflict between heat-inactivated dairy postbiotics and the Codex Alimentarius live-culture standard. Harmonized regulations and characterization standards are critical needs for postbiotic functional food development.

## 1. Introduction

Interest in microbiota-directed functional foods has increased substantially over recent decades, driven by growing consumer awareness of the diet–health relationship, demand for clean-label formulations, and the recognition of the gut microbiota as a key regulator of host metabolism, immune function, and intestinal barrier integrity [1,2]. Within this context, probiotic products have received considerable scientific and commercial attention, as live microorganisms can confer health benefits when administered in adequate amounts [3]. However, their practical application in food systems remains constrained by a fundamental technological limitation: microbial viability must be maintained throughout processing, storage, and gastrointestinal transit, while probiotic efficacy is inherently strain-specific and matrix-dependent [3]. Food systems routinely expose microbial cells to heat treatment, oxygen, acidity, osmotic stress, and extended ambient storage conditions, all of which may compromise probiotic survival and functionality [3,4]. Safety concerns have also been reported in vulnerable populations, including preterm infants, immunocompromised individuals, and patients with impaired gut barrier function, further limiting the scope of universal application [5].

Postbiotics have emerged as a promising alternative or technologically robust complement to live probiotics. According to the International Scientific Association of Probiotics and Prebiotics (ISAPP) consensus statement, postbiotics are defined as a “preparation of inanimate microorganisms and/or their components that confers a health benefit on the host” [6]. Because microbial viability is not required, postbiotics may offer several practical advantages for functional food development, including improved thermal stability, greater compatibility with acidic and low-water-activity matrices, extended shelf life, easier incorporation into processed foods, and reduced dependence on cold-chain logistics [4,6,7]. Their non-viable nature may also reduce certain safety concerns associated with the administration of live microorganisms in high-risk populations [6,7].

Despite this potential, important challenges remain. A major issue is definitional consistency and practical implementation: although the ISAPP framework has provided a clear scientific foundation, many studies in the food science literature still employ cell-free supernatants or crude fermentation filtrates that do not always satisfy strict postbiotic criteria [6,8]. Standardization represents a further unresolved barrier; unlike probiotics, which are routinely quantified using viable cell counts, postbiotics currently lack universally accepted potency assays, compositional benchmarks, and class-wide quality standards, complicating comparisons across studies, batch-to-batch consistency, and regulatory evaluation [7,8]. Furthermore, postbiotic preparations are inherently heterogeneous and may contain inactivated whole cells, cell wall fragments, exopolysaccharides, peptides, organic acids, bacteriocins, enzymes, and other metabolites, each possessing distinct physicochemical behavior, stability profiles, and biofunctional properties [4,8,9].

The current literature is also fragmented across fermentation science, food processing technology, encapsulation research, food safety, and regulatory policy. Recent reviews have highlighted the growing relevance of postbiotics in food preservation, functional food development, and health promotion; however, relatively few integrate the entire pipeline from production and physicochemical characterization through formulation, industrial application, and commercialization [4,7,8,9]. Accordingly, this review examines: (i) the ISAPP definitional framework and its practical limitations; (ii) fermentation-based and non-thermal production strategies; (iii) physicochemical characterization and quality criteria; (iv) formulation and delivery systems, including drying and encapsulation approaches; (v) food applications encompassing biopreservation in meat, seafood, dairy, fresh produce, and fermented foods; and (vi) the global regulatory and commercialization landscape. By synthesizing current evidence across these domains, this review aims to clarify the present status of postbiotics in food systems and identify the key scientific and technological gaps that must be addressed for broader industrial adoption.

## 2. Conceptual Framework, Classification, and Definitional Limitations of Postbiotics

The postbiotic sector has suffered a long history of disjointed and conflicting terminology space. As of 2021, there were at least six different definitions of the term postbiotic published in the peer-reviewed literature, as each included limitations and contradictions which hindered the systematic evidence synthesis, regulatory communication, and coherent health claim substantiation [6,7]. To address this disunity, the International Scientific Association of Probiotics and Prebiotics (ISAPP) organized a panel of experts and came up with a landmark statement of consensus on the definition of a postbiotic as a preparation of inanimate microorganisms and/or their components that confer a health benefit to the host [6]. The definition carries a strictly technical significance in every term: inanimate is a deliberate choice over inactive in order to ensure the absence of cellular viability instead of metabolic dormancy; preparation is meant to be a formulation derived using characterized progenitor strains, generated through a specified matrix and inactivation system, such that the identical technological process would be required to be used to recapitate the same health effect; and it must confer a health benefit [6,7].

The following strict adherence to the ISAPP framework, despite being a landmark step, poses a major practical limitation to food science research, which should be admitted. The most significant restriction is the biomass retention requirement: ISAPP expressly excludes filtrates containing no cellular components and substantially purified metabolites, no matter whether they have been demonstrated to be biologically active, as the definition line must be drawn to avoid the unacceptable classification of chemically synthesized compounds as postbiotics [6,7]. But the prevailing paradigm of experimental research in food biopreservation and functional food studies is based on cell-free supernatants (CFSs) that are centrifuged and filtered fermentation-derived products in which cellular biomass has been removed in part or entirely, acting as convenient postbiotic analogues [8,10]. Omitting CFS-based research would effectively make any functional review of postbiotic food application incomplete, as this type of research forms the bulk of available evidence in the food system. The second unsolved problem is in regard to quantification: unlike the probiotics controlled by ISO 19344 viable cell count standards, there is no internationally agreed upon method of enumerating or characterizing inanimate postbiotic preparations by component classes, a weakness that directly restricts regulatory assessment, standardization of clinical doses, and quality assurance of batches [6,7,11]. Another practical limitation is the requirement of health benefit demonstration which requires clinical trial evidence in the target host; this is not formally fulfilled by the vast majority of food system studies, which demonstrate bioactivity in vitro or in food matrix models [4,12].

**Figure 1:**
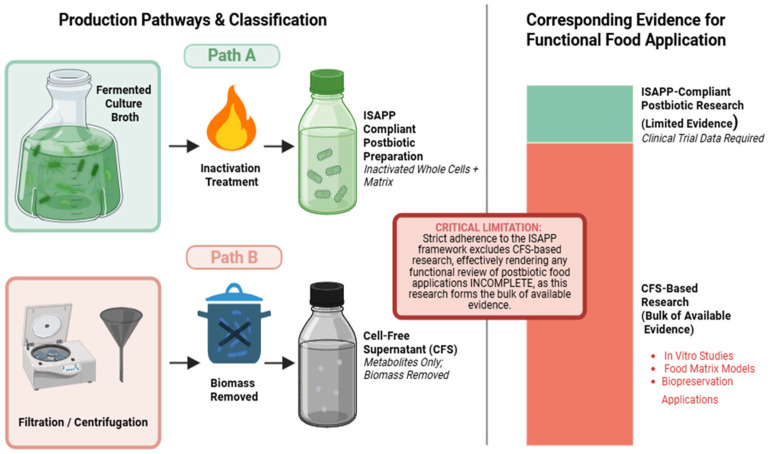
This image illustrates the ways in which postbiotics can be produced according to the ISAPP 2021 consensus framework [6]. Postbiotic production pathways are shown by Path A, where the fermented culture broth is inactivated to produce an ISAPP-compliant preparation of inanimate whole cells co-retained within the fermentation matrix and ISAPP. Path B, which involves filtration or centrifugation to harvest a cell-free supernatant (CFS) that, while lacking co-retained cellular material and is therefore ISAPP non-compliant, constitutes the majority of the functional food literature [7]. ISAPP: International Scientific Association of Probiotics and Prebiotics. CFS: cell-free supernatant.

The production pathways of postbiotics are shown in a simplified form in Figure 1, with the fermented culture broth being inactivated to generate an ISAPP-compliant preparation of inanimate whole cells co-retained within the fermentation matrix (Path A) or the culture-free supernatant (CFS) harvested by filtration or centrifugation (Path B)—the latter constituting the majority of the functional food literature [5,6] (Figure 1).

Postbiotic ingredients represent a very heterogeneous population of bioactive compounds which are grouped in the food science literature into two broad categories depending on the source and physicochemical characteristics: structural or cell-associated components and secreted cellular metabolites [4,12]. These structural elements are inanimate intact cells or paraprobiotics, cell wall fragments (consisting of peptidoglycans, lipoteichoic acids, S-layer proteins, and pili), and exopolysaccharides (EPSs). The secreted metabolites include short-chain fatty acids (SCFAs), bacteriocins, and bioactive peptides that are retained together with cellular biomass in an ISAPP-compliant preparation. More importantly, this dual classification is an abstract organizational system used in the food science literature. The subdivisions themselves are not formally named by ISAPP itself [6,12]. The extraction and inactivation procedures utilized during the manufacturing of the postbiotic directly dictate the final bioactive composition of the postbiotic preparation [13], which is explored in depth in Section 3. With the observance of the above-mentioned definitional limitations, this review uses the wider operational approach of the practice of food science today (which includes not only fully ISAPP-compliant preparations but also CFS-based postbiotic systems) but explicitly defines compliance status where applicable in the context of findings interpretation [7,8].

## 3. Modes of Production, Characterization, and Quality Criteria

### 3.1. Fermentation-Based Production Systems

Conventional fermentation employs lactic acid bacteria (LAB) and *Bifidobacterium* species as the main production organisms, as well as yeasts, including *Saccharomyces cerevisiae* and other species such as *Streptococcus* and *Bacillus*, as the variety of producing organisms reflects the breadth of the range of postbiotic metabolite classes with functional application potential [14,15,16,17]. The biosynthesis of secondary metabolites is determined by the nutritional matrix, and alternative renewable carbohydrates and low-cost agro-industrial by-products, such as distillers’ grains, cheese whey, and microalgal hydrolysates, can support high biomass and metabolite titers at a very low cost in comparison with the conventional commercial media [17,18,19,20,21]. Moreover, coculture of the microbial partners takes advantage of complementary extracellular enzyme repertoires to expand the diversity of postbiotic metabolites, such as bacteriocin-like inhibitory substances (BLISs) and antioxidant phenolics, as exemplars, but the extent and selectivity of these benefits vary by strain and are matrix-specific [16,17]. The optimization of process parameters indicates an underlying trade-off in operation between biomass and metabolite production such that the active control of pH reduces inhibitory osmotic pressure caused by the accumulation of organic acids, and the deliberate introduction of uncontrolled free-fall pH allows the production of defined bioactives such as organic acids, exopolysaccharides, and intracellular polyphosphate fractions with particular metabolites [22,23]. To overcome the substrate inhibition and product toxicity inherent in simple batch systems, the strategic shift across process modes between batch and fed-batch, and ultimately continuous or immobilized fermentation serves as a major scalability lever, especially when combined with in situ product removal (ISPR); in one experiment with *Bifidobacterium animalis* subsp. *lactis HN019*, the ISPR approach improved viable biomass density [21,24,25,26]. This yields an abundant variety of bioactive chemical classes for downstream functional application [27,28].

### 3.2. Thermal Inactivation Methods

The conversion of living cultures into stable postbiotic preparations is done by microbial inactivation. Inactivation must be optimized for full destruction while avoiding loss of functional metabolites because of overheating [20]. Thermal inactivation methods such as pasteurization (60–100 °C), sterilization (121 °C, 15 min), and tyndallization can achieve this but are less useful when thermolabile bacteriocins and phenolic fractions are retained as essential bioactive components of the postbiotic preparation [20,29].

### 3.3. High-Pressure Processing (HPP)

High-pressure processing (HPP) is based on cold pasteurization by the application of high isostatic pressure (100–600 MPa) [15,29]. HPP aims to reach microbial inactivation at a level required by regulators, preserving, at the same time, the structural integrity of thermolabile bioactive fractions, including bacteriocins and phenolics sensitive to the heat degradation commonly present in products after a thermal treatment. Thirumdas and Mudgil also indicated that microbial inactivation by HPP leads to a reduction in cell damage of thermolabile functional metabolites compared to non-thermal heat treatment mechanisms in which cellular metabolism is inactive [30]. Furthermore, Souza and co. highlighted that HPP and pulsed electric fields present the highest level of industrial maturity in the scaling up of these fermentation-based food systems compared to other non-thermal technologies [29] (Table 1).

### 3.4. Pulsed Electric Fields (PEFs)

Highly intense electric field pulses are generated by a pulsed electric field (PEF) considering electroporation effects on microbial membranes at the microscale; in postbiotic production, the application of PEF is double: at sub-lethal intensities, metabolites are released from cell producers by PEF acting as an extraction agent for the recovery of bioactive intracellular substances; at higher intensities or longer treatment times, cell inactivation is achieved by PEF [31,32]. This double role of PEF makes it highly adequate as a metabolite extraction technology or as a lethal inactivation treatment depending on the processing parameters selected, and its high industrial maturity for scaling up in fermented food systems has been proved [29] (Table 1).

### 3.5. Ultrasound

High-intensity ultrasound is capable of creating acoustic cavitation microbubbles that are able to induce mechanical rupture of the microbial cell membranes through the action of intense localized shear forces [15,29]. A primary focus of ultrasound in postbiotic production is the release of intracellular metabolites by membrane disruption, while the phenolic fractions are preserved that are otherwise compromised by heat-based inactivation methods [29]. Thirumdas and Mudgil noted that ultrasound is one part of the new non-thermal technology portfolio, which has already proved its ability to inactivate microbial cells while causing less damage to thermolabile functional metabolites compared to traditional heat treatment [30] (Table 1).

### 3.6. UV Irradiation

UV irradiation is a technique that is specifically designed to destroy microbes by the photochemical destruction of their DNA rapidly; the relevant factor in its postbiotic production is that it acts selectively: UV irradiation leads to inactivation through an indirect, mild effect on the small heat-stable metabolites, including organic acids and bacteriocins, making it applicable for the specificity of metabolite classes whose preservation is the primary formulation goal [32] (Table 1).

### 3.7. Cold Plasma

Cold plasma treatment is a physical method that produces reactive oxygen and nitrogen species, which are the main agents that destroy microbial cell membranes [33]. In postbiotic production, its objective is to kill cells; i.e., the bacterium dies, but at the same time, the polyamine that is sensitive to heat is not damaged by elapsing time due to piloting the heat treatment, which is required for the bioactive species to gain health benefits that are notable in comparison to the conventional method of treatment [33,34]. Thirumdas and Mudgil investigated cold plasma as a non-thermal technology in the postbiotic production suite and confirmed that it is able to destroy microbial cells; it also preserves thermolabile metabolites in comparison to regular heat methods [30] (Table 1).

### 3.8. Supercritical CO_2_ (Sc-CO_2_)

**Figure 2:**
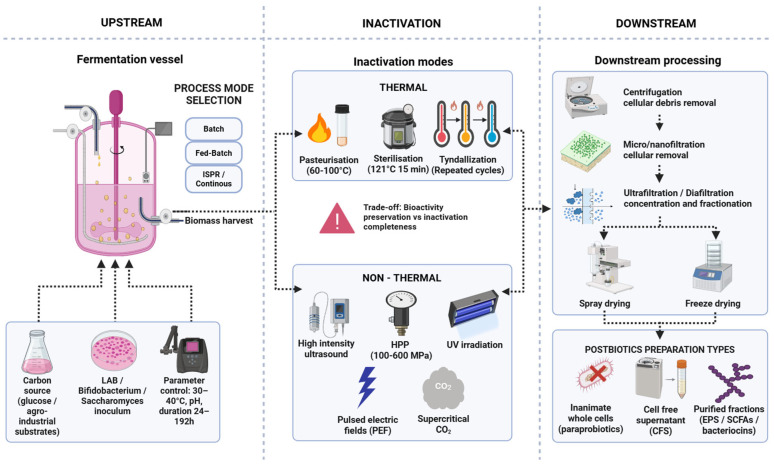
Integrated schematic of the upstream fermentation, inactivation, and downstream processing workflow for postbiotic preparation [20,44]. Upstream fermentation using lactic acid bacteria (LAB), *Bifidobacterium* spp., or *Saccharomyces* spp. is conducted across batch, fed-batch, or in situ product removal (ISPR)/continuous process modes; inactivation is achieved by thermal methods, pasteurization (60–100 °C), sterilization (121 °C, 15 min), or tyndallization or by non-thermal technologies, including high-pressure processing (HPP; 100–600 MPa), pulsed electric fields (PEFs), high-intensity ultrasound, UV irradiation, and supercritical CO_2_ (Sc–CO_2_); downstream processing yields three final preparation types: inanimate whole cells (paraprobiotics), cell-free supernatant (CFS), or purified fractions comprising exopolysaccharides (EPS), short-chain fatty acids (SCFAs), or bacteriocins [24,29,32]. LAB: lactic acid bacteria; ISPR: in situ product removal; HPP: high-pressure processing; PEFs: pulsed electric fields; Sc–CO_2_: supercritical carbon dioxide; CFS: cell-free supernatant; EPS: exopolysaccharides; SCFAs: short-chain fatty acids.

Supercritical CO_2_ or Sc-CO_2_ is a technology platform that depends on the use of high-pressure carbon dioxide as the main absorbable element by the microbial cells that induce their lysis through a rapid deflation [35]. As a biotechnological process element, Sc-CO_2_ is primarily a means of obtaining solvent-free organic materials which have bioactive properties and, simultaneously, of those materials preserving their physicochemical attributes; researchers in the field of postbiotic literature have suggested that Sc-CO_2_ is capable of yielding postbiotic formulations [35,36,37]. Figure 2 shows the overall course of the combination of upstream fermentation, inactivation with heat or cold methods, and downstream processing that is performed through centrifugation, microfiltration, nanofiltration, ultrafiltration, and diafiltration to drying to finally arrive at the postbiotic preparation format. On top of that, Table 1 provides an overview of the key technologies involved in the production, preservation, and delivery of postbiotics in functional foods. It shows the technology’s applications, the bioactive components retained, and the major functional characteristics reported.

**Table 1:** Processing technologies for postbiotic production, stabilization, and delivery.

Technology	Application	Postbiotic Fraction Preserved/Enhanced	Key Outcome	Reference
HPP, PEF, ultrasound, cold plasma (non-thermal)	Microbial inactivation during postbiotic production	Bacteriocins, thermolabile functional metabolites	Review-based evidence: NTTs reduce thermal damage to functional compounds vs. conventional heat treatment	[30]
HPP, PEF, ultrasound, cold plasma	Food fermentation optimization	Heat-sensitive bioactives, phenolics	Review-based evidence: NTTs maintain heat-sensitive compounds in fermented systems; HPP/PEFs have high industrial maturity	[29]
Nanocomposite encapsulation (biopolymer + nanoclay/metal nanoparticles)	Antimicrobial active packaging	Bacteriocins (nisin, pediocin), essential oils	Controlled release; sustained inhibition against foodborne pathogens demonstrated in active packaging	[38]
Spray-drying (maltodextrin carrier)	Fermented *Spirulina platensis* postbiotic encapsulation	Total phenolics, antioxidants, antimicrobials	Maltodextrin encapsulation increases bioactivity vs. uncoated; distinct particle morphologies confirmed by SEM	[39]
CMC film embedding	Bacteriocin incorporation into active packaging	Plantaricin W, enterocin F4-9	Maintained inhibitory efficacy against meat pathogens during refrigerated storage	[40]
Kombucha fermentation (turmeric-enriched)	Functional beverage bioactive enrichment	Phenolics, antioxidants, antimicrobials	Turmeric addition significantly enhanced antioxidant, antimicrobial, and cytotoxic activity profile vs. standard kombucha	[41]
Kombucha fermentation (oolong/yerba mate tea)	Functional beverage characterization	Organic acids, polyphenols, antioxidants	Physicochemical, antioxidant, and sensory properties characterized across tea substrates	[42]
Co-fermentation (*Lp. plantarum* + *S. cerevisiae*)	Sourdough and bread quality enhancement	Mixed LAB + yeast metabolites	Improved bread quality indicators; elevated bioactive compound profiles in fermented water	[43]

HPP = high-pressure processing; PEF = pulsed electric fields; NTT = non-thermal technology; CMC = carboxymethyl cellulose; SEM = scanning electron microscopy; LAB = lactic acid bacteria.

### 3.9. Characterization Methods and Quality Criteria

It is essential to perform physicochemical characterization of postbiotic fractions to set identity, potency, and stability benchmarks of component classes. Unfortunately, there is no internationally agreed method for enumeration or characterization of inanimate postbiotic preparations by component class. This results in limitations in regulatory assessment, clinical dose standardization, and batch quality assurance [6,7]. Table 2 presents major postbiotic component classes and their stability data based on the published literature.

Rahman et al. showed that the antibacterial activity of LAB-derived postbiotic preparations is pH- and temperature-sensitive, becoming inactive at high temperature and during extended storage, as would be expected through degradation of peptides in thermally hostile environments, but this study did not specifically evaluate simulated GI digestion [23]. Jalali et al. confirmed that CFS from *Lactobacillus rhamnosus* and *Limosilactobacillus reuteri* retained measurable antioxidant and antimicrobial activities after a simulated surface-contact challenge on red meat at 4 °C; for topical food preservation applications of this type, GI transit stability is a secondary design criterion rather than the primary functional constraint, given that the postbiotic acts at the food surface rather than the gut interface; this framing reflects the design logic of surface decontamination applications rather than an explicit finding of the cited study [48].

## 4. Formulation and Delivery Modes

To deploy a positive health effect, bioactive fractions must cross the gastrointestinal (GI) barrier and survive enzyme degradation; GI stability constraints where trypsin and α-chymotrypsin decrease proteinaceous postbiotic activity and intestinal bile salts’ ability to completely kill bacteriocin-like activity provide a clear technological imperative of encapsulation when the target of formulation is intestinal [50,51,52]. Aside from providing gastrointestinal (GI) protection, encapsulation also has a second function, to stabilize the efficacy of postbiotics within complex food matrices. Often, postbiotic potency is lower in these food matrices than in the culture media in the laboratory, as the bioactive components bind to or interact with food components. This binding is minimized by encapsulating the bioactive components, as the reactive bioactive fraction is physically isolated and insulated from the matrix interactions [47,53,54]. The selection of platforms is regulated according to three criteria applied: (i) encapsulation efficiency (EE) to target fractions, (ii) controlled release profile to suit the selected site of action, and (iii) compatibility with the receiving food matrix and processing conditions. Table 3 summarizes the performance data of validated encapsulation systems against these metrics.

### 4.1. Drying Technologies

Downstream processing involves centrifugation to isolate cellular biomass and microfiltration and nanofiltration to ensure the removal of all cells after inactivation [44]. These bioactives are then concentrated and partially fractionated using ultrafiltration and diafiltration [24,32]. The stabilization process is completed by drying; lyophilization is a good preservative of biological structures; however, spray drying is a continuous and cost-effective method of industrial microencapsulation but requires protective wall materials to ensure sensitive bioactive fractions are not lost to thermal stress during atomization [20,24,44]. Since the multi-step chromatographic purification process makes purified products very costly to produce, and not commercially scalable, the industry is adopting less processed formulations. Unfractionated crude postbiotic mixtures and cell-free supernatants circumvent the expensive chromatographic procedures with seemingly similar functional and antimicrobial activity to highly purified analogs in food-application trials [8,16,17,32]. Spray-drying is the most industrially scalable encapsulation modality in postbiotic delivery, with the carrier type being the most crucial factor in dictating the preservation of bioactive compounds through drying and storage thereof. Postbiotics in fermented *Spirulina platensis* were encapsulated using maltodextrin, resulting in increased total phenolics, antioxidant activity, and antimicrobial activity compared to those without maltodextrin, with scanning electron microscopy showing clear morphologies of maltodextrin-coated particles in different ratios [39] (Table 1).

### 4.2. Microencapsulation and Biopolymer Matrices

Hydrogel microencapsulation in alginate-based matrices is the most rigorously tested platform to deliver oral postbiotics because of the mild gelation conditions, GRAS status, and pH-responsive swelling behavior. Alginate beads, conjugated with divalent cations, retain their structure at gastric pH, below 3.0, and dissolve in a controlled manner as luminal pH increases, above 6.0, in the distal ileum and colon, releasing encased postbiotic fractions at the selected location of microbiota contact [55]. Alginate-encapsulated postbiotics (*Lactiplantibacillus plantarum*) orally administered as an applied functional food model in a murine colitis model significantly regulated gut microbiota composition, increased colonic SCFA levels, and reduced proinflammatory cytokine expression compared to unencapsulated controls, demonstrating that encapsulation maintained functional bioactivity through simulated GI transit and site [56]. Additional mucoadhesive capabilities and increased intestinal residence time are provided by composite biopolymer systems that include chitosan as an outer layer of coating, although the regulatory status of chitosan as a direct food additive differs across jurisdictions and must be established before commercial formulation [57,58,59]. Leveraging carboxymethyl cellulose (CMC) films as a solid-state delivery system of bacteriocins plantaricin W and enterocin F4-9 shows that bacteriocins embedded in the films retained an inhibitory effect against meat-related pathogens over refrigerated storage periods [40].

### 4.3. Nanoencapsulation: Liposomes and Lipid-Based Carriers

Delivery of bacteriocins and other antimicrobial agents through lipid-based nanoencapsulation systems is beneficial, as they can be protected from deactivation and formed in high local concentrations at the target sites [60]. Nanoencapsulation of the bacteriocin-like substances from *Enterococcus faecalis HY7* in liposomes caused a fourfold increase in the antibacterial activity against vancomycin-resistant Enterococcus faecalis V853 compared to the non-encapsulated preparation [61].

Stimuli-responsive nanocapsules extend this principle to precision colonic targeting, microfluidically fabricated, alginate core-shell microcapsules encapsulating indole-3-propionic acid (IPA), a tryptophan-derived postbiotic metabolite with demonstrated intestinal-barrier-strengthening activity, achieved greater than 85% encapsulation efficiency with less than 8% payload release at gastric pH 1.2, followed by approximately 75% cumulative release in simulated intestinal and colonic fluids within 4–6 h, confirming the selectivity of the delivery window [55]. Liposome-encapsulated nisin at greater than 90% encapsulation efficiency incorporated into casein–gelatin nanocomposite films reinforced with halloysite nanoclay demonstrated sustained antimicrobial activity against *L. monocytogenes*, *B. cereus*, and *C. perfringens* in milk agar food-simulating assays, with a hybrid free-plus-encapsulated approach identified as optimal for controlled interfacial release [38].

**Figure 3:**
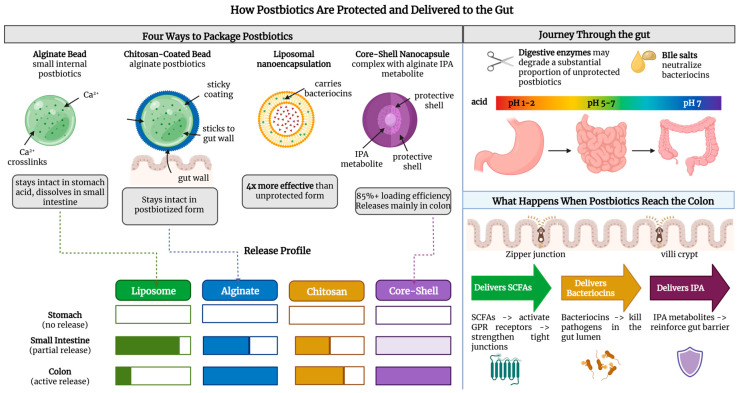
Schematic representation of encapsulation strategies for gastrointestinal protection and site-specific delivery of postbiotic bioactives, depicting four systems, alginate beads (Ca^2+^ crosslinked), chitosan-coated beads (mucoadhesive), liposomes (fourfold greater activity versus unencapsulated form), and core-shell nanocapsules (>85% loading efficiency; preferential colonic release), alongside their comparative gastrointestinal release profiles and functional outcomes at the colonic epithelium, where SCFAs activate G-protein-coupled receptors (GPRs) to reinforce tight junctions, bacteriocins eliminate luminal pathogens, and indole-3-propionic acid (IPA) metabolites strengthen the gut barrier. SCFAs: short-chain fatty acids; IPA: indole-3-propionic acid; GPR: G-protein-coupled receptor; CFS: cell-free supernatant.

A critical formulation challenge specific to functional food applications is that nanoencapsulation systems must remain stable through food processing unit operations (pasteurization, homogenization, freeze–thaw cycling) and retail shelf life periods of 6–18 months; there is not enough evidence available at present to evaluate nanoparticle stability, postbiotic bioactivity retention, and consumer sensory acceptability in a single food product model simultaneously, which constitutes a key translational gap. The encapsulation systems that have been reviewed in publications and how well they performed based on the delivery criteria used are summarized in Table 3 and graphically shown in Figure 3.

**Table 3:** Encapsulation platforms for postbiotic delivery: performance across applied criteria.

Platform	Wall/Shell Material	Target Fraction	EE (%)	Release Trigger & Profile	Key Food-Relevant Outcome	References
Alginate hydrogel beads	Sodium alginate + CaCl_2_	Heat-inactivated *Lp. plantarum* (postbiotic)	Not quantified	pH > 6.0 (colonic dissolution)	Functional SCFA elevation, microbiota modulation, and colitis attenuation confirmed via oral delivery	[56]
Alginate core-shell microcapsule (microfluidic)	Alginate core + chitosan shell	Indole-3-propionic acid (IPA) postbiotic metabolite	>85%	<8% at pH 1.2; ~75% cumulative in SIF/SCF within 4–6 h	pH-triggered colonic-selective release window confirmed; mucoadhesive chitosan layer extends residence time	[55]
Liposome/nanovesicle	Phosphatidylcholine bilayer	Bacteriocins from *Enterococcus faecalis HY7* secretome	Not reported	Not characterized in this study	Liposomal nanoencapsulation achieved a fourfold increase in antimicrobial activity against vancomycin-resistant *E. faecalis V853* compared to the free fraction	[61]
Liposome-nanocomposite film	Casein–gelatin + halloysite nanoclay	Nisin (bacteriocin)	>90%	Sustained interfacial release; hybrid free + encapsulated optimal	Antimicrobial activity against *L. monocytogenes*, *B. cereus*, *C. perfringens* in food-simulating agar	[38]

EE = encapsulation efficiency; SIF = simulated intestinal fluid; SCF = simulated colonic fluid; IPA = indole-3-propionic acid; GRAS = generally recognized as safe.

Fermented functional beverages, including kombucha formats enriched with turmeric [41] and alternative tea substrates [42], represent an emerging liquid delivery matrix for postbiotic metabolites, with co-fermentation of *Lactiplantibacillus plantarum* and *Saccharomyces cerevisiae* yielding fermented waters with enhanced bioactive profiles applicable to sourdough and bread [43] (Table 1).

## 5. Food Preservation Applications

Postbiotics undoubtedly have a promising potential and can be applied to various fields [4,44]. Nevertheless, this review is intentionally limited to food preservation applications, especially biopreservation in meat, fish, milk products, fruits, and fermented food systems, where the main technological difficulty is the introduction of postbiotics into the food matrix itself. Applications in nutraceuticals and other functional food domains are not within the limits of this review despite being promising, and they should be exclusively addressed in separate works.

The biopreservation of these food matrices with postbiotics is a common thread that rests on the utilization of bioactive compounds produced by LAB as clean-label alternatives to synthetic chemical preservatives [10,62] (Table 4).

### 5.1. Meat Systems

In meat systems, CFS from *Lacticaseibacillus rhamnosus* and *L. reuteri* achieved large log reductions in *E. coli* and *S. aureus* on red meat surfaces under conditions relevant to post-rigor meat, with the postbiotic fraction also displaying strong antioxidant and organic acid content [48]. One study extended to active packaging and demonstrated statistically significant changes in mesophilic and psychotrophic counts of spoilage bacteria in minced beef in CMC films using plantaricin W and enterocin F4-9 over 12 days of refrigeration [40] (Table 4).

### 5.2. Seafood

In seafood, fermentation of raw salmon with the organic acid fermentate Verdad N6 plus nisin enhanced control of *Listeria monocytogenes* during refrigerated storage compared to either agent alone [63], a result also consistent with the well-reported synergy of organic acid fermentates with bacteriocins reported in broader postbiotic biopreservation [10,62]. Sodium alginate/chitosan coatings with CFS of *Streptococcus thermophilus* FUA 329 were applied to Pacific white shrimp, and the accumulation of total volatile basic nitrogen (TVB-N) and total viable counts were significantly reduced after 15 days of cold storage, with sensory scores remaining acceptable when compared with control shelf-life limits [64] (Table 4).

### 5.3. Dairy Matrix

In dairy matrices, Silva et al. established the foundational mechanistic basis for bacteriocin-mediated dairy food preservation, while Popović et al. quantified the efficacy of bacteriocin-producing Enterococcus faecium BGZLM1-5 postbiotics in a milk model, achieving up to approximately 63.5% reduction in *L. monocytogenes ATCC 19111* after three days at refrigeration, summarized as a ≥2 log CFU/mL reduction with stable activity across wide pH and temperature ranges [65,66] (Table 4).

### 5.4. Fresh Produce

For fresh produce, Kaya et al. demonstrated that postbiotics made by *Liquorilactobacillus hordei SK-6* decreased *E. coli* and *L. monocytogenes* on ready-to-eat lettuce to a maximum of about 1.6 log CFU/g at 3% of application at concentrations that were considered food-compatible and with no sensory impact [67]. Kanjan and Sakpetch translated antifungal CFS from *L. plantarum* 124 to a Thai ready-to-eat curry product (Kaeng-Tai-Pla-Haeng), extending microbiological shelf life from 7 to 21 days without commercial preservatives, primarily by targeting *Aspergillus flavus* and *Penicillium* spp. surface contamination [68]. Rashid et al. demonstrated that *Enterococcus faecium*-immobilized alginate films incorporating enterocin achieved approximately 3 log CFU/cm^2^ reductions against *Salmonella enterica* on chicken during chilled storage, with in vitro and in vivo safety of the system confirmed [69]. For comparative context, Jin et al. showed that a non-fermentation based composite coating of chitosan, organic acids, and allyl isothiocyanate improved Salmonella safety and extended shelf life in grape tomatoes, serving as a chemical antimicrobial control to which postbiotic surface treatment strategies can be compared [70] (Table 4).

### 5.5. Fermented Foods

In fermented plant systems, Păcularu-Burada et al. selected wild LAB strains that can produce BLIS and antifungal postbiotic compounds in the fermentation of gluten-free sourdough, with metabolites remaining stable following thermal and acidic conditions [71]. Melia et al. characterized the antimicrobial potential of *Pediococcus acidilactici* from Bekasam fermented fish, consistent with the documented range of postbiotic-producing strains from fermented food matrices [72]. Nowak-Lange et al. also determined that postbiotics of *L. pentosus B1* show antibacterial and antibiofilm actions, such as full biofilm inhibition and eradication with higher concentrations, and thermostability; the antibiofilm dimension is specifically applicable to food contact surface decontamination, where planktonic MIC-based measurements alone underestimate the resistance in biofilm states [73] (Table 4).

**Table 4:** Postbiotic biopreservation applications across food matrices.

Postbiotic Format	Food Matrix	Target Pathogen/ Spoilage Organism	Key Quantitative Outcome	References
Organic acid fermentate (Verdad N6) + nisin	Raw salmon	*Listeria monocytogenes*	Improved control during refrigeration; synergy between fermentate and nisin consistent with the postbiotic biopreservation literature	[63]
CFS (*L. plantarum* 124)	Ready-to-eat Thai curry (Kaeng-Tai-Pla-Haeng)	*Aspergillus flavus*, *Penicillium* spp.	Shelf life extended from 7 to 21 days; heat- and protease-stable CFS; no commercial preservatives required	[68]
CFS (*L. hordei* SK-6)	Ready-to-eat lettuce	*E. coli*, *L. monocytogenes*	Up to ~1.6 log CFU/g reduction at 3% application; food-compatible concentration	[67]
CMC film + plantaricin W + enterocin F4-9	Minced beef	Mesophilic and psychrotrophic spoilage bacteria	Statistically significant log-reduction in viable counts over 12-day refrigerated storage	[40]
CFS (*Pediococcus acidilactici*)	Bekasam fermented fish	General antimicrobial spectrum	Antimicrobial potential confirmed; strain-level characterization of postbiotic-producing *P. acidilactici* from traditional fermented fish	[72]
CFS (*L. pentosus* B1)	Food contact surface/model system	Mixed biofilm-forming bacteria	Complete biofilm inhibition and eradication at higher concentrations; thermostability confirmed; antibiofilm dimension extends beyond planktonic MIC assessments	[73]
In situ postbiotics (wild LAB strains)	Gluten-free sourdough	Fungi; spoilage organisms	BLIS and antifungal compounds generated in situ during fermentation; metabolite stability retained after thermal and acid treatments	[71]
CFS (*E. faecium* BGZLM1-5, bacteriocin-producing)	Milk model	*Listeria monocytogenes* ATCC 19111	Up to ~63.5% reduction (≥2 log CFU/mL) after 3 days; activity stable across wide pH and temperature ranges	[66]
*E. faecium*-immobilized alginate film + enterocin	Chicken (surface model)	*Salmonella enterica*	~3 log CFU/cm^2^ reduction; in vitro and in vivo safety confirmed	[69]
Bacteriocins + protective cultures	Dairy products (review)	*Listeria*, *Staphylococcus*, *Clostridium*	Foundational mechanistic basis for bacteriocin-mediated dairy food preservation established	[65]
Sodium alginate/chitosan + CFS (*S. thermophilus* FUA 329)	Pacific white shrimp	Spoilage bacteria; TVB-N accumulation	Significant TVB-N reduction and TVC control during 15-day cold storage; sensory scores acceptable beyond control endpoint	[64]
CFS (*L. rhamnosus*, *L. reuteri*)	Red meat (surface decontamination)	*E. coli*, *S. aureus*	Large log reductions on meat surfaces under post-rigor-relevant conditions; strong antioxidant and organic acid content confirmed	[48]
*L. helveticus* postbiotics	Milk and ground meat models	MDR *S. aureus*, EHEC *E. coli* O15s7:H7	Membrane disruption confirmed as mode of action; dose-dependent killing across milk and ground meat model systems	[47]

CFS = cell-free supernatant; CMC = carboxymethyl cellulose; TVB-N = total volatile basic nitrogen; TVC = total viable count; BLISs = bacteriocin-like inhibitory substances; MIC = minimum inhibitory concentration; MDR = multidrug resistant; EHEC = enterohaemorrhagic.

## 6. Regulatory Landscape and Commercialization of Postbiotic Functional Foods

### 6.1. Global Regulatory Frameworks

No government or international regulatory agency has incorporated the term “postbiotic” into formal regulation for foods and dietary supplements as of 2025, creating a jurisdiction-dependent patchwork of applicable frameworks that food manufacturers must navigate on a product-by-product basis [8,44,74]. In the United States, postbiotic food ingredients most commonly enter the market through the Generally Recognized As Safe (GRAS) designation, which permits ingredients with established safety records to bypass the formal food additive petition process; structure/function claims for postbiotic dietary supplement formats are permissible under DSHEA 1994 without pre-market FDA approval provided claims are truthful, non-misleading, and substantiated by the manufacturer [4,75]. The European Union presents a more demanding two-stage burden: novel postbiotic preparations must first obtain authorization under the Novel Foods Regulation (EU) 2015/2283, a pathway already successfully completed for inanimate *Akkermansia muciniphila* and *Bacteroides xylanisolvens*, before any associated health benefit claim undergoes a separate and additional EFSA authorization process under Regulation (EC) No. 1924/2006, under which the vast majority of probiotic health claim applications submitted to EFSA have historically been rejected [6,7,76,77]. An international regulatory conflict exists related to the development of postbiotic dairy products since the Codex Standard for Fermented Milks (CXS 243-2003) of the Codex Alimentarius Commission requires the presence of live starter cultures, thus product(s) that do not contain viable microbial cells (postbiotic) may not comply with the Codex definition of yoghurt and other fermented milks in jurisdictions that have adopted this standard [6,78]. Japan’s FOSHU system provides the most developed approved-ingredient model globally, permitting authorized active ingredients to carry health function statements once approved, a structurally more accessible framework for postbiotic functional food commercialization than the EFSA dual-authorization pathway [2,6,76]. Table 5 summarizes the major regulatory frameworks relevant to postbiotic functional foods across different jurisdictions.

**Table 5:** Regulatory instruments applicable to postbiotic food preparations across key jurisdictions.

Regulation/Standard	Reference	Postbiotic Relevance	Jurisdiction	Reference
Novel Foods Regulation	EU 2015/2283	Safety evaluation required; *A. muciniphila* and *B. xylanisolvens* authorized as inanimate postbiotics	EU	[7,76]
Nutrition and Health Claims Regulation	EC No. 1924/2006	EFSA authorization required for any health claim; vast majority of probiotic applications rejected	EU	[7,75,77]
GRAS Pathway	21 CFR	Primary US route for postbiotic food ingredient authorization	USA	[4,79]
DSHEA 1994	Public Law 103-417	Structure/function claims permitted; no pre-market approval required for supplements	USA	[75]
Codex Fermented Milks Standard	CXS 243-2003	Requires live cultures in yoghurt; heat-treated postbiotic dairy would not meet this definition	International	[6,78]
FOSHU System	Consumer Affairs Agency (CAA), Japan	Approved-ingredient health function statements; most accessible model for postbiotic commercialization	Japan	[6,76]

GRAS = Generally Recognized As Safe; DSHEA = Dietary Supplement Health and Education Act; FOSHU = Foods for Specified Health Uses.

### 6.2. Labeling, Health Claims, and Food Technology Implications

The most consequential regulatory implication of the ISAPP 2021 definition for food product development is that by embedding a health benefit requirement within the term itself, “postbiotic” functions as an implicit health claim in jurisdictions where claims require pre-authorization triggering the full EFSA dual-authorization burden before the term can appear on any EU product label [6,7]. EFSA’s prior rejection of the generic term “probiotic” as a label claim on the grounds that it implies an unsubstantiated health benefit establishes the interpretive precedent that will apply directly to postbiotic labeling [7,75]. The ISAPP guidance further specifies that substantially purified isolated metabolites do not meet the postbiotic definition and must be designated by their chemical names rather than as “postbiotics”, a precision requirement with direct formulation and compliance implications for manufacturers of bacteriocin- or SCFA-concentrated preparations [6,74]. For food technologists, these constraints practically mandate that postbiotic functional food development proceed either through GRAS or novel food authorization of defined preparations or through clean-label formats where postbiotic fractions are generated in situ during fermentation without requiring the term to appear on the label [30].

### 6.3. Commercialization Barriers

The most fundamental barrier to postbiotic commercialization is the complete absence of widely adopted, cross-class standardized production protocols, potency assays, batch-consistency benchmarks, or minimum efficacious dose frameworks, meaning no regulatory authority can evaluate quality equivalence between manufacturer submissions, and no manufacturer can reliably substantiate a health claim to the standard required for authorization [2,8,44]. Section 3 has set non-thermal inactivation technologies as the best of both by proving them superior to thermolabile bioactive preservation; however, further regulatory validation is necessary to ensure compliance with industrial safety before they can be accepted by authorities as an alternative to conventional thermal processing that creates a direct barrier between best-practice food science and commercially authorizable manufacturing methods [30]. The postbiotic industry is currently experiencing a high growth rate, but industry commercialization is hindered by poor consumer understanding of probiotic–postbiotic products, lack of retail differentiation, and the lack of sanctioned health claims that could allow evidence-based product positioning [2,44].

### 6.4. Documented Regulatory and Commercialization Research Gaps

**Figure 4:**
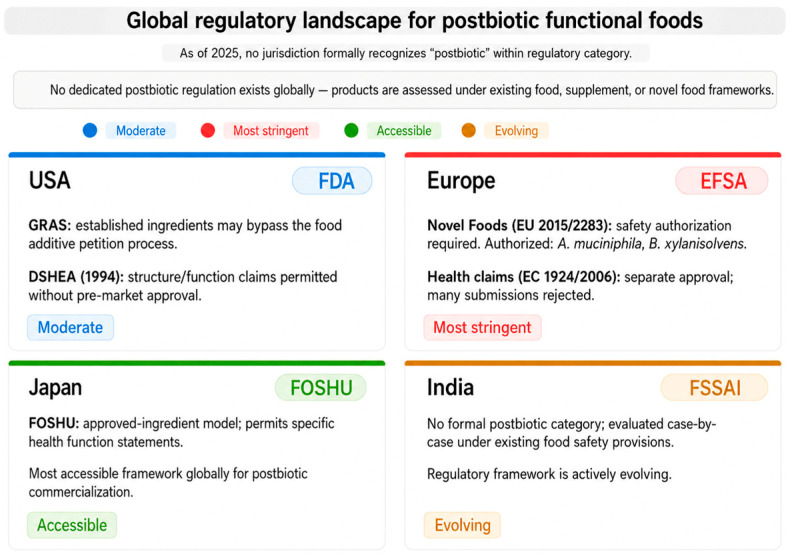
Overview of the global regulatory landscape applicable to postbiotic functional food products across four major jurisdictions as of 2025, with no jurisdiction formally recognizing “postbiotic” as a distinct regulatory category [4,6,7]. Products are assessed under existing food, supplement, or novel food frameworks, with overall stringency rated as moderate (USA), most stringent (Europe), accessible (Japan), and evolving (India). FDA: Food and Drug Administration; EFSA: European Food Safety Authority; FOSHU: Foods for Specified Health Use; FSSAI: Food Safety and Standards Authority of India; GRAS: Generally Recognized as Safe; DSHEA: Dietary Supplement Health and Education Act.

Three priority gaps collectively define the translational bottleneck between postbiotic food science and commercial market entry. First, no government or international regulatory agency has incorporated the term “postbiotic” into formal regulation for foods or dietary supplements as of 2025, leaving manufacturers to navigate fragmented probiotic, novel food, and food additive frameworks not designed for inanimate microbial preparations—an ambiguity that will persist until ISAPP’s definitional framework is formally adopted within at least one major regulatory system [7,8,44]. Second, no widely adopted, cross-class standardized potency assay, quality benchmark, or minimum efficacious dose framework exists for any category of postbiotic preparation, fundamentally preventing comparative efficacy demonstrations and batch-consistency verifications that regulatory authorization requires [2,8,44]. Third, the active conflict between the CXS 243-2003 live-cultures requirement and the defining characteristic of postbiotic dairy preparations and inactivated microbial material remains unresolved [78], with no Codex harmonization process underway that would create a classification pathway for heat-treated postbiotic dairy products in the large number of jurisdictions where the Codex standard is applied as national regulation [2,6,8]. The jurisdiction-specific regulatory frameworks, their governing bodies, and relative stringency levels for postbiotic functional food approval are summarised in Figure 4.

## 7. Future Outlook and Research Priorities

The trajectory of postbiotic functional food science will be shaped by progress across three interconnected domains: production standardization, regulatory harmonization, and advanced delivery system validation.

The most pressing near-term priority is the development of component-class-specific standardized protocols for postbiotic production, characterization, and potency determination. As no cross-class benchmarks currently exist for batch consistency, minimum efficacious dose, or quality equivalence across manufacturer submissions, no regulatory authority can meaningfully evaluate postbiotic health claim applications, and no clinical study can generate generalizable dose–response data [2,8,44]. Internationally recognized reference standards that are analogous to those currently used on probiotics under ISO 19344 will be required to substantiate health claims with credibility and will necessitate a collaborative effort by ISAPP, Codex Alimentarius, and national food safety authorities [6,11].

As for non-thermal inactivation technologies, Figure 2 identified them as superior for thermolabile bioactive preservation, representing the most significant near-term production opportunity, but their commercial adoption is contingent on regulatory validation frameworks confirming industrial safety compliance for HPP-, PEF-, and cold plasma-derived postbiotic preparations [29,30]. EU and USA regulatory bodies have not yet established technology-specific guidance in non-thermal inactivation in the production of postbiotics, and it is this gap that needs to be filled to unlock the quality potential of these technologies at the industrial level [4].

In the delivery systems domain, the key translational gap is the lack of any research that concurrently describes nanoparticle stability, bioactivity retention, and consumer sensory acceptability throughout commercially realistic shelf lives of 6–18 months; it is necessary to bridge this gap before nanoencapsulation platforms can be transitioned to food product deployment [38,55]. Further studies are needed to focus on standardized accelerated shelf-life testing regimes of lipid-based and biopolymer nanoencapsulation systems on processing-relevant food matrices.

At the regulatory frontier, the formal incorporation of the ISAPP postbiotic definition into at least one major regulatory framework, whether through EFSA scientific opinion, FDA GRAS guidance, or FSSAI novel food provisions, would provide the legislative foundation for authorized health claims and generate the commercial certainty needed to attract sustained investment in postbiotic product development [2,7,76]. Simultaneously, omics-driven approaches, including metabolomics, proteomics, and metagenomics, offer the analytical resolution required to fully characterize postbiotic preparations, elucidate mechanisms of action at the molecular level, and generate the structure-function evidence that regulatory health claim substantiation demands [80,81].

## 8. Conclusions

This review offers a holistic overview of the pipeline for postbiotic development, including definitions, food preservation, and regulatory challenges. The ISAPP 2021 consensus definition—a preparation of inanimate microorganisms and/or their components that confers a health benefit to the host—is a major milestone in the definition of terms, but its retention of biomass formally excludes cell-free supernatant (CFS)-based products that represent the bulk of the evidence in food science, a dichotomy that needs to be acknowledged in an operational definition rather than avoided. Non-thermal processing technologies, such as high-pressure processing (HPP), pulsed electric fields (PEFs), ultrasound, cold plasma and supercritical CO_2_, prove superior to traditional thermal processing in retaining heat-sensitive bacteriocins and phenolic fractions, making them the preferred method of production; yet, the lack of globally harmonized potency testing and quality standards across postbiotic component sub-classes is the most pressing gap in both regulatory evaluation and dose generalizability in clinical trials. Microencapsulation in alginate hydrogels, nanoencapsulation in liposomes, and spray-drying with maltodextrin carriers have all been shown to effectively protect postbiotic bioactives during gastrointestinal transit and food matrix challenges, but no published research has yet confirmed the triple benefits of nanoparticle stability, bioactivity retention, and consumer acceptability over commercial storage lifetimes. In meat, fish, milk, fresh fruits and vegetables, and fermented food products, postbiotic formulations delivered substantial reductions of major food pathogens (*Listeria monocytogenes, Salmonella* spp., *E. coli* O157:H7), demonstrating their scientific plausibility as clean-label alternatives to chemical preservatives. The term “postbiotic” remains unrecognized in any major jurisdiction as of 2025, and three areas represent priority translational bottlenecks: the lack of cross-class standardized quality criteria; the lack of formal regulatory adoption of the ISAPP definition; and the unresolved incompatibility between the Codex Alimentarius live-culture standard (CXS 243-2003) and postbiotic dairy formats rendered heat-inactivated. The establishment of class-specific protocols for standardization of components, adoption of omics-based approaches for postbiotic characterization, and regulatory harmonization—with at least one major jurisdiction formally adopting the ISAPP definition—are the key priorities for promoting the commercial development of postbiotic functional food products.

## Figures and Tables

**Table 2:** Physicochemical characterization and thermal stability of major postbiotic component classes.

Postbiotic Fraction	Producer Strain/Source	Characterization Method	Key Stability Finding	Reference
EPS	*Streptococcus thermophilus* CC30	TGA, FTIR, monosaccharide composition	Degradation onset above pasteurization range; stable molar mass	[45]
Kefiran (EPS)	Kefir grain microbiota (cow, goat, sheep milk)	TGA, NMR, SEM	Extraction method determines MW retention; solvent extraction preserves high-MW fraction	[46]
CFS (bacteriocin + organic acid fraction)	*L. helveticus*	Chemical characterization, antioxidant assays, cell-based safety	pH- and temperature-stable in milk and ground meat models; antibacterial against MDR MRSA and *E. coli* O157:H7	[47]
CFS (mixed bioactive fraction)	*L. rhamnosus*, *L. reuteri*	Antioxidant/antimicrobial assays, red meat surface application	Antioxidant and antimicrobial activity retained after surface application at 4 °C; GI stability secondary for topical preservation design	[48]
Mixed postbiotic preparation	LAB (multiple strains)	Preparation-method comparative study (CFS, heat-killing, enzymatic + sonication)	Preparation method significantly modulates bioactive compound profile and antimicrobial efficacy; standardization essential	[49]
CFS/BLIS fractions	LAB (multiple strains)	Thermal, pH, and storage stability assays	Activity stable up to 121 °C, pH 3–11, and storage at 4–20 °C; elevated temperature and prolonged storage identified as primary activity-loss drivers consistent with peptide degradation	[23]
Spray-dried fermentate	Fermented *Spirulina platensis*	Spray-drying + maltodextrin encapsulation	Maltodextrin encapsulation increases total phenolics, antioxidant capacity, and antimicrobial activity; SEM confirms distinct particle morphologies	[39]

TGA = thermogravimetric analysis; FTIR = Fourier-transform infrared spectroscopy; NMR = nuclear magnetic resonance; MW = molecular weight; MDR = multidrug resistant; MRSA = methicillin-resistant Staphylococcus aureus; GI = gastrointestinal; CFS = cell-free supernatant; BLIS = bacteriocin-like inhibitory substances.

## Data Availability

No new data were created or analyzed in this study. Data sharing is not applicable to this article.
